# Infection prevention and control: knowledge, determinants and compliance among primary healthcare workers in enugu metropolis, south-east nigeria

**DOI:** 10.1016/j.infpip.2022.100214

**Published:** 2022-04-27

**Authors:** Casmir N. Ochie, Elias C. Aniwada, Eloka K. Uchegbu, Thaddeus C. Asogwa, Chika N. Onwasoigwe

**Affiliations:** a Department of Community Medicine, University of Nigeria Teaching Hospital, Ituku-Ozalla, Enugu, Nigeria; b Department of Community Medicine, College of Medicine, University of Nigeria, Nigeria; c Department of Community Medicine, ESUT College of Medicine, Enugu, Nigeria

**Keywords:** Infection prevention and control, Primary healthcare workers, Knowledge, Determinants, Administrative control, Enugu

## Abstract

**Introduction:**

Healthcare workers have lost their lives in significant numbers in the discharge of their duties as a result of a breach in Infection Prevention and Control (IPC) procedures. The increasing incidence of emerging and re-emerging diseases complicates this burden. Adequate IPC includes administrative, environmental and personal protective control measures. This study assessed the knowledge, determinants and compliance to IPC among primary healthcare workers in Enugu Metropolis.

**Methodology:**

A cross-sectional study was done using a semi-structured interviewer-administered questionnaire. A multi-stage sampling technique was used to select 300 eligible Health Care Workers in Primary Health Care facilities. Analyses were done using IBM-SPSS version 23. Ethical approval was obtained from the Health Research Ethics Committee of UNTH Enugu.

**Results:**

The majority of the respondents were Community Health Extension Workers (CHEWs) or Community Health Officers (CHOs) and nurses 122 (40.7%), 197(65.7%) were female; with a mean age of 39.86 ± 9.62 years. Only 254(84.7%) of the respondents had previous IPC training and 82(27.3%) of them had good knowledge of IPC. Needle-stick injury was identified as a source of occupational exposure to infections amongst 185(61.7%). A majority, 244(81.3%) could not correctly identify all the moments of hand washing.

**Conclusion:**

The demonstrated poor level of knowledge and compliance to IPC demands more research to unravel this existing gap. However, these conditions can be improved by training the workers on IPC.

## Introduction

Infection control is a combination of measures aimed at minimizing the risk of infection transmission within populations. [1] It entails standard precautions and transmission-based precautions. Any breach in these infection control precautions facilitates and sustains the chain of transmission of infection from patients to health care workers, to other patients and visitors. [2] Poor IPC knowledge and compliance could be associated with untold consequences among patients. Such consequences include prolonged duration of hospitalization, increased severity of the primary illness, increased cost of care with unquantifiable impact on their quality of life and that of their families. [3] Healthcare workers are an indispensable component of the health system and they play cardinal roles in IPC, which invariably contribute to the quality of patient care and management.

The magnitude of the problem of poor knowledge of IPC is particularly noticed in an environment where basic infection control measures are usually lacking or non-existent in health facilities. [4] Hospital-acquired infections are among leading causes of death in the US [5] and contribute to the shortage of human resources for health. [4] Severe Acute Respiratory Syndrome (SARS), recognized as the first deadly emerging and transmissible disease by the World Health Organization in the 21^st^ century, was noted to have easily spread through close hospital contact with infected persons. [6] In fact, as SARS spread, it became obvious that many countries lacked the necessary infrastructure, facilities, equipment and trained personnel to provide appropriate infection control measures. [6] A hospital can provide a favourable transmission environment for the spread of infections; especially in facilities having staff with poor knowledge of IPC. [7] Gaps have been identified in the knowledge and compliance of IPC among healthcare workers by some authors. [8] With the rising rates of tuberculosis, hospital-acquired infections and other emerging and re-emerging diseases like SARS, Middle East Respiratory Syndrome (MERS) and COVID-19 internationally, it is necessary to take precautions to prevent infectious disease transmission in healthcare settings. The transmission of infections is possible in health facilities providing a wide range of health services (as found in the Primary Health Care (PHC) Centres). Hence, the need to ascertain the level of IPC knowledge among staff, because the determinants and availability of administrative control measures remains a significant step to break the chain of transmission of such infections.

Infection control in healthcare facilities places special emphasis on standard and additional (transmission-based) precautions. [9] Several scholars have worked on IPC within different levels of healthcare systems globally, [[10], [11], [12], [13]] and more locally within Nigeria. [[14], [15], [16]] Very few investigated IPC knowledge at PHCs. Most authors agreed that healthcare workers in Nigeria were knowledgeable and compliant with standard precautions, [17] which is just an aspect of IPC. Others still held the view that the practice of IPChad remained very poor in healthcare settings. [18] However, the Ebola outbreak of 2014 could not be easily forgotten as it reawakened the inevitable need to observe IPC strategies, [18] not just amonghealthcare workers but everybody in the West Africa sub-region (including Nigeria). This then underscored the fact that the responsibility of IPC should be that of everybody. Important components of infection control programmes include basic measures for infection control, (i.e. standard and additional precautions); education and training of health care workers; protection of health care workers, (e.g. immunization against some diseases like hepatitis); identification of hazards and minimizing risks; routine practices essential to infection control such as aseptic techniques, use of single-use devices, appropriate reprocessing of instruments and equipment, antibiotic usage, management of blood/body fluid exposure, handling and use of blood and blood products and sound management of medical waste [9] However, infection control comprises four major aspects which include administrative measures, environmental measures, personal protective measures and waste disposal measures [19].

Having noted the consequences of poor knowledge of IPC among the PHC workers with no study on the administrative control aspect of IPC in Nigeria, this study aimed to identify the level of knowledge of IPC among the PHC workers, its determinants and the presence of IPC administrative measures in PHC facilities.

## Methods

This study was conducted in Enugu State, located in the South-East geopolitical zone of Nigeria. There are 17 Local Government Areas (LGAs) in Enugu state: 12 rural LGAs and 5 urban LGAs. Enugu state has an estimated population of 3.3 million and a total land area of 7,618 sq. km with well-developed coal mining, commercial, financial and industrial centres. [20] Inhabitants of the state engage in agriculture, commerce (trading) and a good number of the urban dwellers are civil servants. [21] Enugu State has four tertiary hospitals, > 500 Public Health facilities and > 3000 private health facilities. However, this study was done in the Enugu metropolis which is made up of three local government areas (LGA): Enugu North, Enugu East and Enugu South.

This was a cross-sectional study of 300 selected primary health care workers in 30 facilities. The minimum sample size was determined using the sample size determination formulae for descriptive cross-sectional study [22] $n=\frac{z^{2}\mathrm{pq}}{d^{2}}$ with “p” being a proportion of respondents with good knowledge of infection prevention and control from a previous similar study, 77.2% (0.772) [23] carried out in Enugu, South-East Nigeria, q is the complementary probability (1 – p) = 1–0.772= 0.228 and d is the desired precision of the study set at 0.05. This gave a minimum sample size of 297 rounded up to 300 respondents. A two-stage sampling technique was used to select the participants. The first stage involved the selection of 10 PHC facilities in each of the three LGAs (since the number of facilities in each LGA is approximately equal) by simple random sampling using the balloting method. The second stage involved the selection of 10 PHC workers from each of the 10 selected PHC facilities in the LGA using a simple random sampling technique by balloting. The facilities and respondents were selected from the staff list provided by the state ministry of health.

An interviewer-administered, semi-structured questionnaire with the following sections: sociodemographic variables, knowledge of standard precautions of IPC, transmission-based precautions of IPC and availability of IPC administrative programme structures were used.

The researcher and four trained research assistants (who were rigorously trained for two days) collected the data over four weeks, during October–November 2019. Quantitative variables like age and years of practice were summarized using means and standard deviation. Categorical variables like knowledge, marital status, etc were summarized using frequencies and percentages and analyses were done using the computer software IBM-Statistical Package for Social Sciences, IBM SPSS version 23. The outcome variables include the proportion of those with good knowledge and the proportion of the facilities that comply fully with administrative provisions of IPC programme structures and facilities for IPC. The knowledge level was determined by assigning values of 1 and 0 respectively for good and poor knowledge for each of the knowledge variables. An overall good knowledge score was assigned to those who scored at least 50% of the total score whereas scores less than 50% were considered poor knowledge. The test statistics employed in this study include chi-square and the level of statistical significance was set at *P*-value ≤ 0.05. Ethical approval for the study was obtained from the Health Research Ethics Committee (HREC) of the University of Nigeria Teaching Hospital (UNTH), Ituku-Ozalla, Enugu. Also, verbal consent was obtained from all the participating respondents whereby their confidentiality was guaranteed by reassurance and using anonymous questionnaires.

## Results

A total of 300 copies of the questionnaire were distributed and retrieved; giving a response rate of 100%. The mean age (± standard deviation) of the respondents was 39.86 (±9.62 years) and the majority of respondants were aged 31–40 years. Females constituted the majority(65.7%) of respondants. Most of the workers had tertiary education (84.3%) and 205(68.3%) were married. Considering the occupation of workers, CHEWS/CHO made up 40.7% of respondants and 208(68.7%) of respondents had ≤10 years of practice/experience. Only 254(84.7%) reported ever having IPC training, among which 241(80.3%) had IPC training at most 10 years prior this study. See Table I.

**Table I:** Socio-demographic characteristics of PHC workers in Enugu Metropolis

Socio-demographic variables	Frequency	Per cent
Age in years		
20–30 years	65	21.7
31–40 year	104	34.7
41–50 years	84	28.0
51–60 years	47	15.6
**Mean ± SD**	39.86 ± 9.62	
**Sex**		
Male	103	34.3
Female	197	65.7
**Educational level**		
Secondary and below	47	15.7
Tertiary	253	84.3
**Marital status**		
Single	72	24.0
Married	205	68.3
Others∗	23	7.7
**Professional cadre**		
Nurse	121	40.3
CHEW/CHO	122	40.7
Others	57	19.0
**Years of practice/experience**		
≤10	208	68.7
11–20	62	20.7
>20	30	10.6
**Mean ± SD**	10.04 ±7.77	
**Had IPC training**		
Yes	254	84.7
No	46	15.3
**If yes (category of last training period) previous years**		
1–10	241	80.3
11–20	13	4.3

Overall, 218(72.7%) of these healthcare workers had poor knowledge (i.e < 50% of the knowledge scores) of IPC. Exactly 271(90.3%) did not know that considering the potential for transmission of infectious agents in patients constitutes part of the concept of standard precautions and 285(95.0%) had poor knowledge of the use of puncture-resistant containers as a standard precaution practice, whereas 185(61.7%) had good knowledge that needlestick injuries/sharps could be potential sources of occupational exposure to infections. Considering the moments of handwashing, a majority (60.7%) did not know that hands should be washed before any direct contact with patients, in-between patient contacts (74.7%) and after touching body fluids (81.3%). However, the majority knew that hands should be washed immediately after removing gloves (69.7%). The majority (66.0%) did not also know that standard precautions should be applied for all patients irrespective of the clinical presentation. Two hundred and sixty-eight (89.3%) had poor knowledge of the fact that all blood-tinged body fluids required standard precautions. See Table II. The majority of the healthcare workers (55.0%) considered that hepatitis B infection was an important patient factor in deciding the use of Personal Protective Equipment (PPE) and 13.7% of respondents also had good knowledge of indications for when anti-retroviral drugs should be given as Post Exposure Prophylaxis (PEP). See Table II, Table III**.**

**Table II:** Knowledge on Infection Prevention and Control measures among PHC workers in Enugu Metropolis

Variables	Correct	Incorrect
	Freq(%)	Freq(%)
Concept of infection prevention and control (IPC)		
Hand washing before and after direct contact with a patient	219(73.0)	81(27.0)
Consideration potential for transmission of infections	29(9.70	271(90.3)
Cough etiquette	38(12.7)	262(87.3)
Safe injection practices	31(10.3)	269(89.7)
Wearing PPE	47(15.7)	253(84.3)
Safe needle and sharp handling	83(21.2)	152(38.8)
**Infection Prevention and Control against blood and body fluid**		
Use of gown and protective apron	17(5.7)	283(94.3)
Universal precaution instituted-policy	125(41.7)	175(58.3)
Use of gloves Surgical masks and face shields	24(8.0)	276(92.0)
Recap needles immediately after use	87(29.0)	213(71)
Use of mouthpiece and resuscitation bags	41(13.7)	259(86.3)
Use of puncture resistant container	15(5.0)	285) (95.0)
**Potential ways of occupational exposure to infections**		
Needlestick injury/sharp	185(61.7)	115(38.3)
Splash on the eye	93(31.0)	207(69.0)
Inhalation	36(12.0)	264(88.0)
Talking to patients	17(5.7)	283(94.3)
Touching patients	29(9.7)	271(90.3)
**Moments of hand washing**		
Before any direct contact with patients	118(39.3)	182 (60.7)
Between patient contact	76(25.3)	224(74.7)
Immediately after removing gloves	209(69.7)	91 (30.3)
After touching body fluids	56(18.7)	244(81.3)
**Conditions where IPC are followed**		
Blood borne pathogens eg HIV	129(43.0)	171(57)
Tuberculosis	75(25)	225(75)
Patients with skin infections	31(10.3)	269(89.7)
All patients	102(34.0)	198(66.0)
**Body fluids that require IPC**		
Blood	128(42.7)	172(57.3)
Vaginal fluid	63(21.0)	237(79.0)
Blood tinged body fluid	32(10.7)	268(89.3)
Saliva in dental procedures	23(7.7)	277(92.3)
All of the above	104(34.7)	196(65.3)

**Table III:** Knowledge of Infection Prevention and Control among PHC workers in Enugu continued

Variables	Yes	No
	Freq(%)	Freq(%)
Important patient factors in the decision to use PPE		
HIV/AIDS patients	42(13.7)	258(86.3)
Hepatitis B patients	165(55.0)	135(45.0)
Signs and symptoms of patients	19(6.3)	281(93.7)
All patients	91(30.3)	209(69.7)
**Post Exposure Prophylaxis is given only to HIV negative healthcare workers**	256(85.3)	44(14.7)
**Duration of PEP is for 4 weeks**	256(85.3)	44(14.7)
**Overall knowledge**		
***Good***	*82(27.3)*	
***Poor***	*218(72.7)*	

There is a statistically significant association between years of practice (experience) and overall knowledge (χ2 =8.469, *P* = 0.037) but no statistically significant association between having training on IPC and overall knowledge (χ2 =2.533, *P* =0.149). The professional occupation and years of practice were found to be determinants of good knowledge. The odds of a nurse having good knowledge of IPC is 2.50 times higher than that of other professional PHC workers (orderlies, ward maids, and laboratory and pharmacy technicians) (AOR 2.5; 95% CI 1.256–4.969) whereas the CHEW/CHO are shown to have odds 2.0 times higher than the odds of other PHC professionals (orderlies, ward maids, and laboratory and pharmacy technicians) in having good knowledge of IPC (AOR 2.0; 95% CI 1.023–3.967). Similarly the odds of respondents that have worked for ≤10 years having good IPC knowledge were about 3 times higher than the odds of other respondents with >20 years' practice (AOR 2.88; 95% CI 0.73–17.25) and those who had worked for 11–20 years had odds of good IPC knowledge about 2 times higher than that of those with practice more than 20 years (AOR 1.89; 95% CI 0.66–10.63). See Table II, Table III.

**Table IV:** Determinants of knowledge on IPC among PHC workers in Enugu metropolis

Variable	Overall knowledge		*χ*^*2*^ test (*P*-value)	AOR (95% CI)
	Good	Poor		
**Age in years**				
20–30 years	23(35.4)	42(64.6)		
31–40 year	24(23.1)	80(76.9)	2.624	NA
41–50 years	14(16.7)	70(83.3)	(0.453)	
51–60 years	8(34.8)	43(91.5)		
**Sex**				
Male	30(29.1)	73(70.9)	0.254	NA
Female	52(26.4)	145(73.6)	(0.614)	
**Educational level**				
Secondary and below	16(34.0)	31(66.0)	1.263	NA
Tertiary	66(26.1)	187(73.9)	(0.261)	
**Marital status**				
Single	25(34.7)	47(65.3)	2.619	NA
Married	51(24.9)	154(75.1)	(0.270)	
Others^a^	6(26.1)	17(73.9)		
**Professional cadre**				
Nurse	20 (16.5)	101(83.5)	8.024	2.50 (1.26–4.97)
CHEW/CHO	36(29.5)	86(70.5)	(0.018)	2.02 (1.02–3.97)
OTHERs^b^	9(15.8)	48(84.2)		1
**Years of practice**				
≤11	65(31.3)	143(68.8)	8.345	2.88(0.73–17.25)
11–20	15(24.2)	47(75.8)	(0.015)	1.89 (0.66–10.63)
>20	2(6.7)	28(93.3)		1
**Training on IPC**				
Yes	57(22.4)	197(77.6)	0.583	NA
No	8(17.4)	38(82.6)	(0.445)	

a widows, widower, separated.

b health attendants, security, cleaners.

Concerning the availability and compliance to administrative IPC measures among primary healthcare workers, the majority of them reported not having the following: Infection Prevention and Control Committee (IPCC) (73.3%), IPC focal person (84.0%), signages to aid movement (76.7%), IPC policy (72.3%) and an institutional IPC meeting (71.0%). Of those reporting having IPC meetings, 35.6% said the frequency of their meeting was weekly. A majority also reported non-availability of the following in their facilities: visible standard operating procedures (75.7%), IPC plan duly signed (78.3%) and IPC plan/checklist in place (76.3%), see Table II, Table III.

**Table V:** Reported IPC administrative programme/structures in the PHC facilities in Enugu Metropolis

Variables	Yes	No
	Freq(%)	Freq(%)
**Presence of IPC committee/team**	80(26.70)	220(73.3)
**Presence of IPC focal person**	48(6.0)	252(84.0)
**Availability of conspicuous Signages to aid movement**	70(23.3)	230(76.7)
**Availability of IPC policy**	83(27.7)	217(72.3)
**Organization of IPC meeting**	87(29.0)	213(71.0)
**Frequency of the IPC meeting**	Freq	%
Daily	2	2.3
Weekly	31	35.6
Monthly	28	32.2
Annually	10	11.5
Not regular	16	18.4
**Availability of SOP on the walls**	73(24.3)	227(75.7)
**Any report or document on accidental injuries for appropriate actions**	191(63.7)	109(36.3)
**Availability of IPC plan duly signed**	65(21.7)	235(78.3)
**Availability of IPC plan checklist in place**	71(23.7)	229(76.3)

## Discussion

Knowledge of IPC among PHC workers can make or mar the healthcare practice at this basic level of the healthcare system. Previously published literature acknowledges that healthcare workers have above-average knowledge of standard IPC precautions. [16,[24], [25], [26]] A study in Nigeria documented that the healthcare workers' knowledge of standard IPC precautions was good, [8,16] but similar to our findings, studies in Enugu, South-East Nigeria, [23] and in Nepal on infection control among PHC workers, showed that 22% and 18% respectively had the correct knowledge of IPC. [13] In contrast, similar studies in Plateau State, Northcentral Nigeria and Egypt revealed fairly high levels of knowledge of IPC amongst PHC workers. [3,7,8,16] Health care workers should be equipped with requisite knowledge, skills and attitudes for good infection control practice. The reason for this dissimilarity could be attributed to the inclusion of doctors in those studies, as they were carried out in tertiary healthcare facilities where IPC structures were better established. Inclusion of doctors (trained academically in infection pathophysiology and pathogen transmission) and other healthcare worker groups could lead to this discrepancy in reported results. Doctors were not included in this study.

The implication of these findings could result in increased morbidity and mortality among their patients, vulnerability to antimicrobial resistance and increased referral to higher levels of the healthcare system since the majority of these PHC workers did not exhibit good IPC knowledge.

On the concept of standard precautions as a component of IPC, the majority of our respondents, 90.3% did not know that the potentials for transmission of infectious agents should be considered in all patients and this constituted part of the concept of standard IPC precautions. An implication of this could be increased incidence of hospital-acquired infections (HAI) among patients and primary healthcare workers would have higher chances of acquiring infections from patients. These study participants had a low-risk perception of infection transmission dynamics.Furthermore, there would be higher chances of cross-infection from one patient to another.

A significant proportion ofrespondents had good knowledge of the fact that needlestick injuries/sharps could be potential sources of occupational exposure to infections. This showed that they would be more careful while handling sharps and needles even though knowledge does not translate to good performance consistently. What could not be established was their knowledge failure to appropriately use the puncture-proof container having acknowledged the hazardous nature of the sharps in a healthcare setting.

Considering moments of handwashing, more than half of these respondents could not correctly identify the circumstances for hand washing but they knew that hands should be washed immediately after removing gloves. This could imply a reduced risk of cross-contamination and auto-infection among the workers because hand hygiene had been regarded as the most effective way of infection control and prevention, even though knowledge does not always transmit to good practice.

A majority of these healthcare workers did not know that standard precautions should be observed for all the patients. Most of them were ignorant of the fact that all blood-tinged body fluids required standard precautions. This could be very risky as these body fluids were the vehicles of infectious agents and hence not applying standard precautions to them could increase the chances of more occupationally acquired diseases and the spreading of such, might sustain Healthcare Acquired Infections (HAI). Regarding the concept of standard precaution as a component of IPC, a significant proportion had poor knowledge of this concept with exclusion of hand washing where most of them correctly identified hand washing as a component. These findings contrasted a similar study done in Enugu where the authors reported that many of their respondents had good knowledge of these components. [16] This discrepancy could be explained partly by the fact that the study dealt with standard precautions and most of their study respondents were trained in standard precaution which is one of the elements of IPC. More so, in contrast to this observation, was another study done in North-Central Nigeria where a higher proportion of the healthcare workers were noted to exhibit good knowledge of the concept and its components. [27] This could be explained by the fact that the sample size and scope of that study was higher than that of this study. They included both private and public hospitals which invariably had more doctors and nurses that carry out surgical operations and services that were more complex and complicated than that carried out in primary healthcare facilities. Moreover, these study areas being higher in healthcare level also served as referral centres where infection prevention and control knowledge and practice should be higher.

The findings in this study regarding potential occupational exposures to infection showed that the majority of respondents had good knowledge of a source of exposures to infection being needle stick injury. This was similar to many research findings regarding standard precautions internationally and locally. [10,16,28,29] On indications for post-exposure prophylaxis (PEP), almost all respondants had adequate knowledge of this. This was in keeping other research findings. [16,30].

This work also revealed that nurses were more likely to have overall good knowledge than other professionals (orderlies, ward maids, and lab and pharm technicians). Surprisingly, the years of practice did not influence the overall knowledge. These findings were inkeeping with the outcome of a related study where staff cadre was found to influence good IPC knowledge. [18] The implication is the that nurses and CHEWS/CHOs had more knowledge of IPC. This was understandable because the CHEWs and CHO were largely the staff of PHCs and of course, were more in contact with patients and perhaps with relatively higher risk perception. It would be expected to be associated with higher indices of suspicion for infectious contact in dealing with patients.

Non-institution of an IPC committee within these PHC facilities as reported in this research work, absence of IPC focal persons, no IPC policy nor IPC meetings, absence of IPC plan, non-availability of IPC checklist and non-availability of conspicuous signage to aid movement within the facilities were similar to the findings in South African study on administrative and environmental measures of IPC where only 20% of the facilities reported having IPC plans/checklist in their facilities and 50% per cent of the facilities were noted to have IPC focal persons. [11] However, this finding was not in agreement with that of a study in Jos Plateau state on IPC where half of the respondents reported attending IPC meetings/training [7] and another 6% was reported in Pakistan study on IPC as attending IPC meeting. [31] This could be explained by the fact that healthcare care workers had not considered this aspect of the compliance to IPC as important as other aspects like environmental management, standard and transmission-based precautions.

This study had some limitations. The study tool was self-constructed and prone to bias. However, this tool was pre-tested and validated by the authors. More so, good knowledge of IPC might not translate to good behaviour or practice nor a positive attitude to HAI. A further study would be beneficial in unravelling these associations. The small sample size of this study could also affect the generalizability of the findings even though it is still a representative sample. There will be a need to further ascertain the reasons for the inadequate administrative component in our PHC facilities. Much more insight could be sought in the administrative component of IPC in PHCs perhaps through qualitative studies.

## Conclusion

Knowledge of IPC among healthcare workers is of importance to enhance a healthy working environment. A few of these respondents had good knowledge of IPC and their age, professional cadre, and years of practice were significantly associated with the overall good knowledge. The non-availability of the IPC administrative measures in most of the facilities is an indication of poor political commitment to IPC in the PHCs. There is a need for workers' continuous training and the provision of administrative measures on an escalated scale to ensure a reduction in HAI.

## Conflict of interest statement

The authors have no conflicts to declare.

## Funding

No funding was received for this study.

## Ethics and informed consent

Ethical approval was obtained from the Health Research Ethics Committee of UNTH Enugu. Informed consent from the respondents was obtained after detailing the objectives of the study to them. Participants were given the freedom to withdraw from participating if they so wish.
